# The association between spike technique and injuries in competitive volleyball players—a pilot study

**DOI:** 10.3389/fspor.2026.1737436

**Published:** 2026-02-03

**Authors:** Markus Tilp, Carmen Pusch, Alexandre I. A. Medeiros, Björn Wieland, Yannick Prosch, Karen Zentgraf, Isaac Kneuhbuhl, George Giatsis

**Affiliations:** 1 Department of Human Movement Science, Sport and Health, University of Graz, Graz, Austria; 2 Master Program in Physiotherapy and Functioning, Federal University of Ceara, Fortaleza, Ceara, Brazil; 3 Institute of Physical Education and Sports, Research Group in Biodynamic Human Movement, Federal University of Ceara, Fortaleza, Ceara, Brazil; 4 Movement and Exercise Science, Institute of Sports Sciences, Goethe University Frankfurt, Frankfurt, Germany; 5 Skill Acquisition Specialist & Consultant at TORQVB, Lawnsdale, CA, United States; 6 Department of Physical Education and Sport Science, Aristotle University of Thessaloniki, Thessaloniki, Greece

**Keywords:** bow & arrow, circular technique, overuse injury, shoulder injury, spike technique, volleyball

## Abstract

**Background:**

Different spike techniques in volleyball may vary in performance and shoulder loading, potentially influencing injury risk. However, no previous study has systematically examined the association between spike technique and shoulder injuries in competitive volleyball players.

**Methods:**

An online questionnaire was distributed via coaches to competitive volleyball players from Austria, Brazil, Germany, Greece, Portugal, and the USA between December 2024 and February 2025. The survey collected demographic data, spike technique (bow & arrow or circular), self-reported shoulder injuries or symptoms, and related treatments. Coaches classified players’ spike techniques based on provided descriptions. Group differences were analyzed using Chi-square tests (p < 0.05).

**Results:**

A total of 175 players (90 females, 85 males; mean age 24.3 ± 9.4 years) participated in the study. The bow & arrow technique was most common (71.4%), followed by circular (25.7%). Overall, 32% of the players reported shoulder injuries and 44.7% reported shoulder symptoms. No significant differences in injury or symptom frequency were found between techniques, either overall or within genders (*p* > 0.05). In females, the circular technique showed lower injury (18.2% vs. 31.2%) and symptom (36.4% vs. 51.6%) rates descriptively compared to bow & arrow, but without statistical significance. Males showed very similar rates for the techniques (injury: 39.1% vs. 34.4%; symptoms: 39.1% vs. 42.6%).

**Conclusion:**

The present data do not demonstrate a clear advantage of either spike technique with respect to shoulder injuries or symptoms. Gender-specific trends towards a greater injury risk for women using bow & arrow technique warrant further investigations. Future studies should increase sample size, include a broader range of competitive levels, and also integrate objective biomechanical and medical assessments.

## 1 Introduction

The spike is one of volleyball's most decisive skills for both men and women, serving as a primary predictor of match success (1–3). Multiple spike techniques are used in elite play (4). Prior research indicates that these techniques differ in ball velocity and the mechanical stress imposed on the shoulder (5). Although some have speculated that certain techniques may reduce shoulder injury risk, this relationship has not been formally tested. However, considering the high prevalence of shoulder pain and injury among volleyball athletes (6), such an analysis is of critical importance.

Epidemiologic studies report that overuse shoulder injuries account for approximately 19% of all overuse injuries in volleyball (7), and nontraumatic shoulder pain has been reported in 40% of high school volleyball players, with few taking time off to recover; longer duration of competitive play was significantly associated with increased risk (8). Shoulder issues are highly prevalent among elite indoor players, with 67% reporting complaints during the season and over a quarter (27%) experiencing problems severe enough to limit training or performance (9). These rates are consistent with more general data from overhead sports, where repetitive dominant-arm motion and long-playing history are recognized risk factors for shoulder pathology (10).

From a sports-medical perspective, the repetitive high-velocity overhead motion of spiking, often exceeding 40,000 attacks per athlete annually, subjects the glenohumeral joint and surrounding soft tissues to considerable cumulative stress (7, 11). Overuse injuries such as rotator-cuff tendinopathy, subacromial impingement, scapular dyskinesis, biceps–labral pathology (including SLAP lesions), and glenohumeral internal-rotation deficit are common in this population (12).

A volleyball spike consists of sequential phases: approach, jump, preparatory wind-up, cocking, explosive acceleration, and follow-through. While the acceleration and follow-through phases of the arm are relatively uniform, the arm-swing preparation (preparatory wind-up & cocking phase) varies widely (13–15). Observational and motion-capture studies identified at least two dominant arm-swing styles: the traditional “bow & arrow” and an alternative “circular” pattern, each with distinct kinematics and shoulder loading profiles (5). Although some investigators propose that the circular swing may reduce exposure to injurious shoulder positions, clinical evidence is lacking. During volleyball attacks, shoulder flexion combined with maximal external rotation during elevation of the spiking arm has been identified as a high-risk movement for the glenohumeral joint. Humerus flexion beyond 90° induces high risks for irritation and subsequent injury of the supraspinatus tendon and related structures. These kinematic features are especially pronounced in the bow & arrow technique (5). Moreover, unlike the circular technique, the bow & arrow technique involves a change of direction at the end of the cocking phase, characterized by high decelerations and accelerations that impose high forces in the shoulder joint (4).

In this context, sex-related differences warrant consideration. Epidemiologic studies in beach volleyball—where similar arm-swing mechanics are used—show higher rates of shoulder surgery and diminished shoulder function in women compared with men (16). Motion analyses also indicate sex-based preferences in arm-swing style, with women more often employing so-called snap or straight-arm patterns and men more often adopting a circular swing (2, 17). Additional kinematic differences have been documented between indoor and beach volleyball and between male and female jump mechanics (18, 19). These findings suggest that sex may influence both the distribution of spike techniques and the risk of shoulder injury.

In summary, the high forces generated during the cocking and acceleration phases of the spike subject the shoulder to repetitive tensile and compressive loads comparable to those experienced by baseball pitchers and tennis players (20, 21). Although the later phases of the arm motion are relatively consistent, the wind-up and cocking phases display distinct technical variations—most notably the bow & arrow and circular arm swings (5, 15). Understanding how these variations relate to shoulder health is essential for developing evidence-based injury-prevention strategies and coaching practices.

This study aimed to examine the relationship between spike technique and the occurrence of shoulder injuries or symptoms in competitive indoor volleyball players. A secondary aim was to examine the specific relationship in males and females. We hypothesized that athletes using the circular arm-swing technique would report fewer shoulder injuries or symptoms than those employing the bow & arrow technique and that this would be similar in males and females.

## 2 Method

To collect data on players’ spike technique and shoulder injuries or symptoms in competitive volleyball, we developed an online questionnaire using LimeSurvey. The original version was created in German and subsequently translated into English by a native-speaking researcher with volleyball experience. The questionnaire was distributed via coaches of national league teams in Austria, Brazil, Germany, Greece, Portugal, and the USA. Coaches were informed about the purpose of the study and asked to forward the questionnaire to their players. To minimize inaccurate self-assessment of spike technique, coaches were also provided with detailed descriptions of the bow & arrow and circular techniques (see supplementary material “Coaches information”). They were asked to assess each player's spike technique and share the classification with the players. In accordance with Seminati et al. (5) but in contrast to other previous studies (1, 2, 17), we only distinguished two spike techniques, bow & arrow and circular. Due to their kinematic similarity, the different bow & arrow sub-types (low and high) and the straight technique were defined as bow & arrow while the snap and circular would be defined as circular. The reason for this decision was to allow comparisons with the work by Seminati et al. (5) and expected low numbers of some techniques (e.g., straight). Furthermore, we expected to avoid incorrect assessments by this simple definition in only two different techniques that should be easy to distinguish. The questionnaire was available between December 2024 and February 2025. It included questions about the players’ demographics (e.g., gender, age, volleyball experience, …), spike technique (bow & arrow or circular), shoulder injuries or symptoms, and any therapies received. The full questionnaire is provided in the supplementary material. Descriptive data is presented in absolute and relative values for the overall group as well as separately for male and female athletes. Group differences (e.g., bow & arrow vs. circular technique) were analyzed using Chi-square tests (SPSS). In case the number of cases was <5 for a cell, Fisher's exact tests were conducted. Effect sizes of Chi-square tests are given by Cramér's V (22) and interpreted according to Lee (23). The level of significance was set at 0.05.

All participants and coaches were informed about the aim of the study, without being informed about the specific hypotheses. Players and coaches provided informed consent through the online questionnaire. The study was conducted in accordance with the Declaration of Helsinki and approved by the Ethics Committee of the Aristotle University of Thessaloniki.

## 3 Results

A total of 175 players (90 females, 85 males) from six countries completed the questionnaire correctly and were included in the analysis. The average age was 24.3 ± 9.4 years. Participants came from Germany (25.7%), Austria (21.1%), Greece (18.9%), Brazil (17.1%), Portugal (12.6%), and the USA (4.6%). On average, players had 12.7 ± 8.4 years of volleyball experience. Most participants competed at either the national (89.7%) or international level (8.6%), while 1.7% reported playing at a regional level. All playing positions were represented: 68 outside hitters, 35 middle blockers, 30 opposite hitters, 30 setters, and 12 liberos.

**Figure 1:**
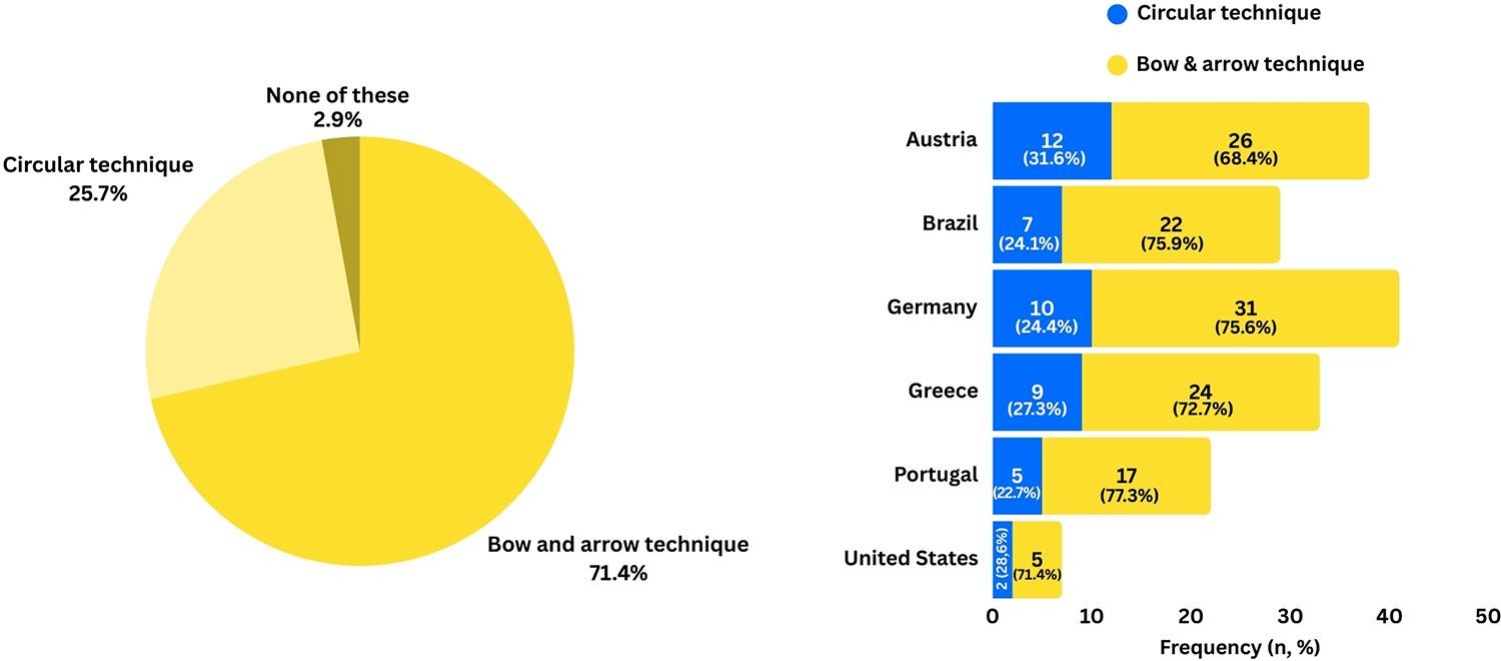
Overall distribution of spike techniques (left panel) and within different countries.

The majority of players (94.7%) reported being aware of the different spike techniques. The bow & arrow technique was the most commonly used (71.4%), followed by the circular technique (25.7%), while 2.9% indicated using neither of these techniques. The distribution of spike techniques varied by country, with the lowest proportion of circular technique users in Portugal (22.7%) and the highest in Austria (31.6%) (see Figure 1). However, Fisher exact test (2-sided) revealed no significant difference between countries (*p* = 0.94). There was no significant difference in spike technique distribution between genders (25.6% of females and 27.4% of males used the circular technique, *χ*² = 0.71, *p* = 0.79, Cramér's V = 0.02).

**Figure 2:**
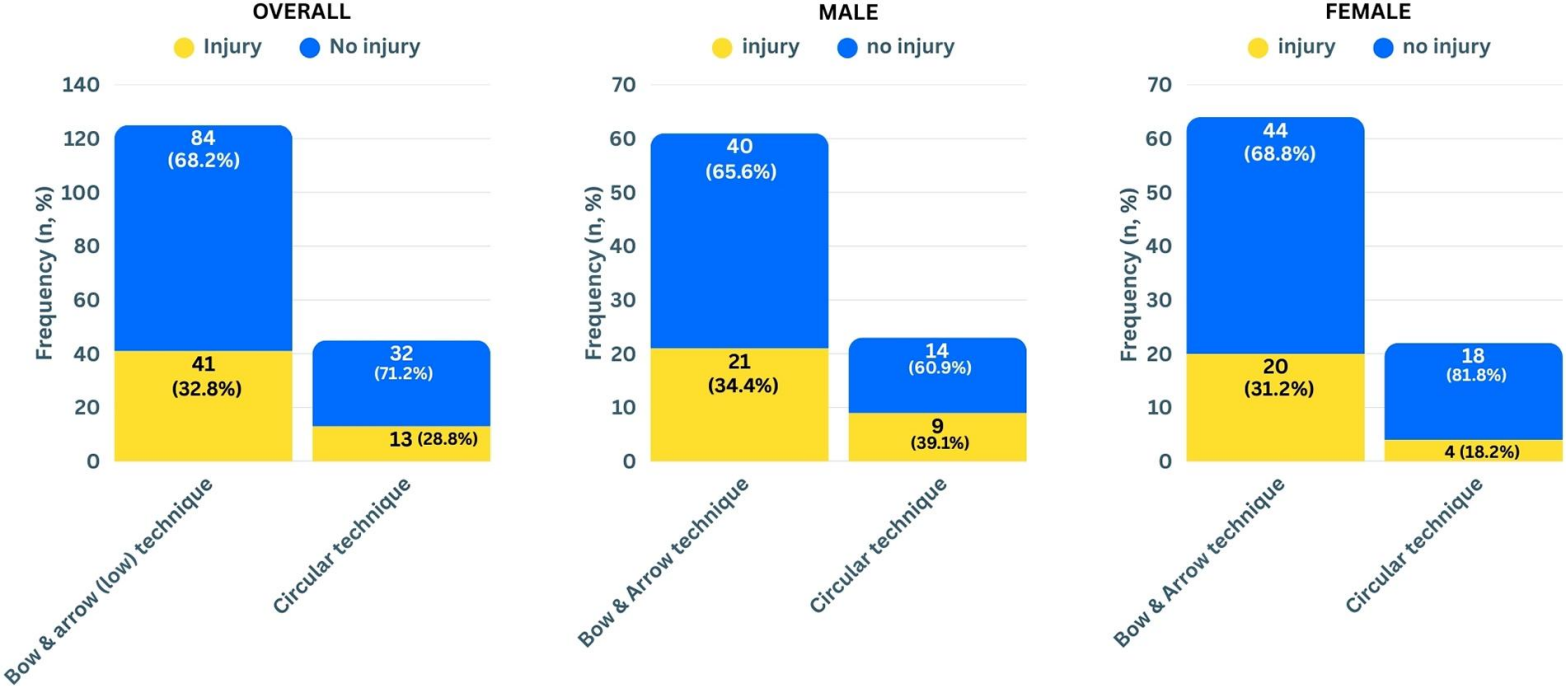
Frequency of shoulder injuries related to spike technique for all participants (left panel, *p* = 0.63), males (middle panel, *p* = 0.69), and for females (right panel, *p* = 0.24).

Overall, 54 (32%) of the players reported that they had shoulder injuries including nine (5%) that needed surgery. The most common injuries were biceps tendonitis (14) and a rotator cuff injury (13), followed by impingement (8), shoulder instability (8), labral tear (5), and supraspinatus rupture (3). There was no significant difference in the frequency of injuries between genders (females 27.8%, males 36.5%, *χ*² = 1.195, *p* = 0.27, Cramér's V = 0.08). In addition, there was no significant difference in injury frequency between the spike techniques (bow & arrow 32.8%, *χ*² = 0.234, circular 28.9%, *p* = 0.63, Cramér's V = 0.04). When analysed for the different genders (Figure 2), the injury frequency between the different techniques was neither significantly different for females [bow & arrow 31.2%, circular 18.2%, Fisher's exact test (two sided), *p* = 0.24, Cramér's V = 0,13] nor males (bow & arrow 34.4%, circular 39.1%, *χ*² = 0.161, *p* = 0.69, Cramér's V = 0.04).

**Figure 3:**
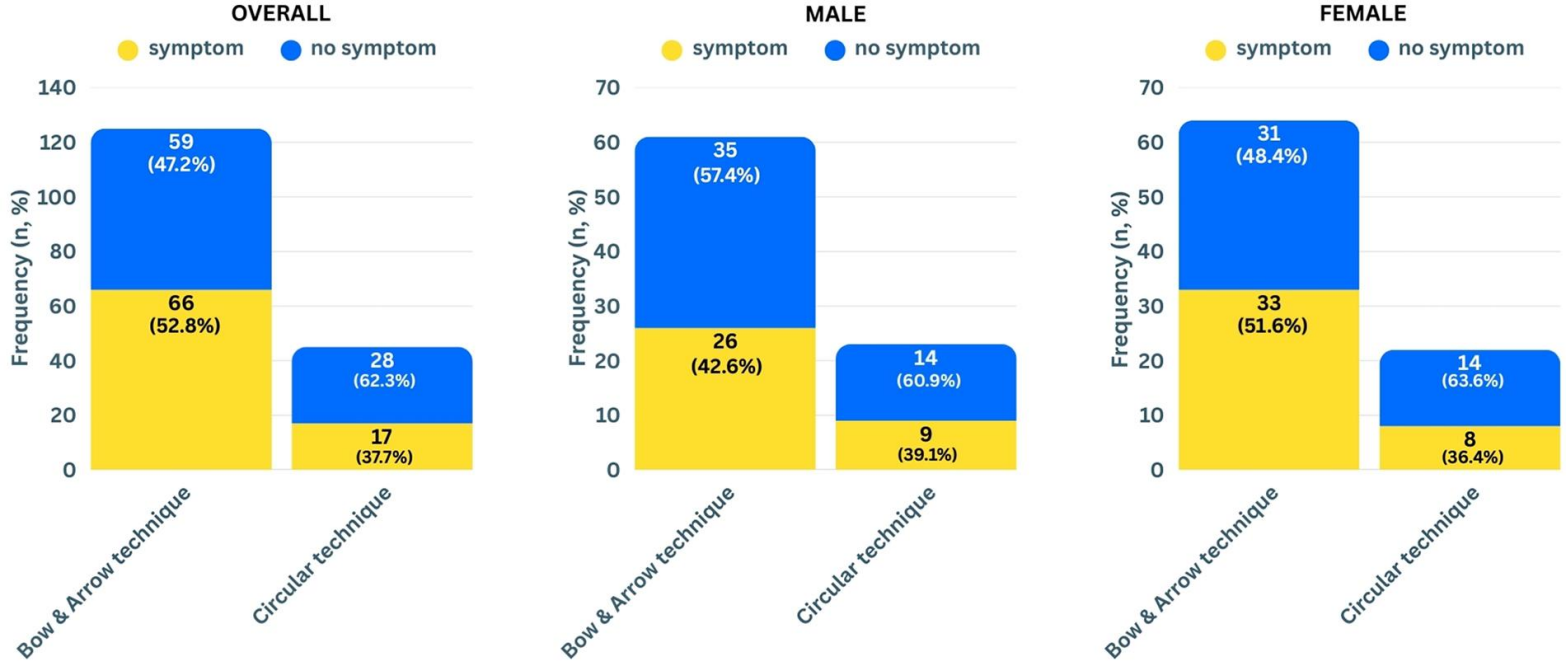
Frequencies of shoulder symptoms related to spike technique for all participants (left panel, *p* = 0.28), males (middle panel, *p* = 0.77), and females (right panel, *p* = 0.22).

Shoulder symptoms were reported by 44.7% of the players, with pain being by far the most frequently mentioned complaint (60), followed by clicking noise (5), loss of strength (5), and shoulder stiffness (3). The frequency of symptoms was neither significantly different between genders (females 47.7%, males 41.7%, *χ*² = 0.62, *p* = 0.43, Cramér's V = 0.06) nor between techniques (bow & arrow 47.2%, circular 37.7%, *χ*² = 1.188, *p* = 0.28, Cramér's V = 0.08). When analysed separately for the genders (Figure 3), symptom frequency was neither significantly different for females (bow & arrow 51.6%, circular 36.4%, *χ*² = 1.52, *p* = 0.22, Cramér's V = 0.13) nor for males (bow & arrow 42.6%, circular 39.1%, *χ*² = 0.84, *p* = 0.77, Cramér's V = 0.03).

Among the total sample, 54.1% of players performed a shoulder training routine. Within the group of injured players 74.1% had such a routine, while among the non-injured players 44.8% reported having one. The difference was statistically significant (*χ*² = 12.7, *p* < 0.01, Cramér's V = 0.27). Overall, 13.9% of the players took pain killers and 19.4% used Kinesiotape because of shoulder injuries/symptoms.

## 4 Discussion

This study is the first that provides data about shoulder injuries and their association to spike technique in competitive volleyball players. A high ratio of 32% of the players who answered our online questionnaire reported shoulder injuries. However, in contrast to our hypothesis we did not observe a significant difference with a negligible effect between players that use bow & arrow compared to those who use the circular spike technique. Similarly, while almost half of the players (44.7%) reported shoulder symptoms, which is comparable to ratios found in previous studies [40%, (8)], the frequencies were also not significantly different with a negligible effect between players that use bow & arrow compared to those who use circular spike technique.

In our sample, the bow & arrow technique was used most frequently (71.4%), followed by the circular technique (25.7%), and other techniques (2.9%). This differs from the data from Seminati et al. (5) of competitive Italian volleyball players where 52% used a circular technique (there named alternative technique) and 48% the bow & arrow technique (there named traditional technique). Furthermore, these numbers are also different from data on Giatsis et al. (2) from elite indoor volleyball players, where the circular technique was the most common in both men (68.2%, snap + circular) and women (62.3%, snap + circular), with bow & arrow techniques being less frequent (2). We assume that differences are due to the performance level of the players which might be somewhat lower in our sample. The frequency of circular technique(s) in our sample is more similar to results reported in beach volleyball, where 73.3% used bow & arrow techniques and 26.6% used circular techniques (17).

The injury (32%) and symptom (44.7%) rates in our sample (24.3 years, 12.7 years of volleyball experience) are similar to previous findings that provide a wide range of shoulder problem prevalence. Clarsen et al. (31) reported a prevalence of 16% shoulder problems and 5% substantial shoulder problems in 65 boarding school pupils (∼17 years,∼5 years of experience) that had daily volleyball training. Almost half (46%) of top-level male volleyball players (*n* = 59, 27.6 years, ∼9 years of experience) from the first English division suffered from shoulder pain (24). Reeser et al. (25) reported that approximately 60% of recreational volleyball players (*n* = 422, 21.3 years, 7.8 years of experience) had a history of shoulder problems. Lajtai et al. (16) even reported that ca. 63% of elite beach volleyball players (*n* = 84, 28 years, 12 years of experience) suffered from pain in their shoulders during an FIVB beach volleyball tournament. In contrast, only 9.1% of male (*n* = 99, 23 years) and 7.3% of female (*n* = 82, 22 years) Slovenian 1st and 2nd division players reported previous shoulder injuries (26).

When comparing the results by gender, no significant differences were found in the frequency of shoulder injuries or symptoms. Interestingly, in females the frequency of shoulder injuries was lower in the circular technique group (18.2%) compared to the bow & arrow group (31.2%), although this difference did not reach statistical significance (*p* = 0.37) and a weak effect. A similar, non-significant trend with a weak effect was observed for shoulder symptoms, with fewer females using the circular technique reporting symptoms (36.4%) compared to those using bow & arrow (51.6%). These tendencies could point toward a possible protective effect of the circular technique in female players, e.g., due to anatomical or body composition differences. However, the relatively small subgroup size, especially among circular technique users, limits the meaningfulness of these trends. Future studies with larger female samples and including biomechanical analyses to objectively determine spike technique could help clarify whether these patterns are consistent and meaningful*.* In male players, the results showed no tendency in direction of a technique: the circular technique group had slightly higher injury (39.1% vs. 34.4%) but slightly lower symptom (39.1% vs. 42.6%) rates compared to bow & arrow users, again without statistical significance and negligible effect.

More than half of the players (54.1%) performed a shoulder routine training program. However, this was significantly different with a moderate effect between players with (74.1%) or without (44.8%) a shoulder injury. This is reasonable as players with shoulder problems are more likely to perform a shoulder routine because of their pain. Although, according to a recent review (27), the effect of shoulder injury prevention programs is neither well investigated nor fully understood, there is some evidence that such programs lower the risk of shoulder problems by 28% in overhead athletes (28). Hence, there would be a potential to prevent shoulder problems for healthy volleyball players by including such programs in their training routine. Another countermeasure against shoulder problems are pain killers. In our sample, 13.9% of the athletes took pain killers which is low compared to data published on elite (∼30%–60%) or recreational (∼30%–90%) athletes (29). In contrast, almost 20% of the athletes reported to use kinesiotape, probably as an alternative to medical drugs. However, the effect of kinesiotaping on injuries is still unclear (30), without any studies investigating its effects in overhead athletes.

A major strength of this study is the relatively large and diverse sample size for this research topic. To our knowledge, this is one of the largest datasets to date combining self-reported spike technique and shoulder health in competitive volleyball players (*n* = 175). Unlike many previous studies that were restricted to single teams, single countries, or elite-only cohorts, our sample covers a broad spectrum of athletes, both male and female, all main playing positions, and various competition levels from regional to international and players from different countries.

This study is not without limitations. First of all, the information provided by the questionnaire is subjective. Although we informed the coaches about the different techniques and provided movement descriptions and respective examples, we cannot rule out that some of the coaches did not assess the spike technique correctly and recommend objective video or 3D biomechanical analysis in future studies. Furthermore, the information about injuries were not given by medical doctors but the players themselves and could therefore be mistaken. However, even if the players made mistakes regarding the type of injury, we firmly believe that the players are aware of their injuries and symptoms. Furthermore, we cannot be sure if the participating players are representative for all competitive volleyball players. It might be that players with an injury/symptom were inclined in participating or even not participating in the study due to their experience with the injury. In theory we have approached a great number of players within the first leagues in six different countries. Estimating 10 teams in the first league for both genders in six countries would lead to approx. 120 teams á 12 players and ultimately sum up to 1,440 players. With 175 players that participated in the study, we would have reached only about 12% of all eligible players. This restricted the opportunity to observe significant effects, especially in the subgroup analyses in both genders and impeded the application of more complex multivariable models to identify different sources of injuries/symptoms. However, as known to the authors, this is still the highest number of competitive volleyball players that provided information about their spike techniques and shoulder injuries/symptoms. Our sample also includes twelve liberos and one could argue that they do not spike as frequently and might bias the results. Therefore, we have performed an additional analysis excluding data from liberos which, however, led to the same result with neither significant differences in injuries (*p* = 0.33, V = 0.08) nor symptoms (*p* = 0.28, V = 0.09) in relation to spike technique. Last but not least, we provide only information about the association between spike technique and shoulder injuries/symptoms but no causal relationship. However, it seems hardly feasible to undertake a randomized controlled trial to investigate a possible causal relationship.

## 5 Conclusion

The data from the present study do not reveal a clear advantage or disadvantage of the bow & arrow or circular volleyball spike techniques with respect to shoulder injuries or symptoms. While certain gender-specific tendencies were observed, such as a lower injury and symptom rate in female circular technique users, these differences were not statistically significant and must be interpreted with caution due to limited subgroup sizes.

Future research should aim to increase the number of participants. Expanding the sample and integrating objective biomechanical assessments could provide a more precise understanding of the potential relationship between spike technique and shoulder health in volleyball players.

## Data Availability

The raw data supporting the conclusions of this article will be made available by the authors, without undue reservation.
