# Supplementary material for: The association between spike technique and injuries in competitive volleyball players—a pilot study
Source: Front Sports Act Living. 2026 Feb 3;8:1737436. doi: 10.3389/fspor.2026.1737436 (PMC12909513; doi:10.3389/fspor.2026.1737436)
Supplement: Supplementary file 1 [file Datasheet1.pdf]

TRAINER INFORMATION

Thank you for participating in our study!

As part of my master's thesis at the University of Graz, this study forms an integral part of an international collaboration aimed for exploring the link between spiking techniques and shoulder injuries in competitive volleyball.

For this study your players will complete a questionnaire with some general and volleyball specific questions by themselves except for question number **10**.
It is marked with a **red** heading saying:
Please ask your trainer to answer this question.

Then we need your expertise to classify the spiking technique of each player in your team.
Analyze the players while spiking and report them the results, which they should choose in their questionnnaire.

To help you a little bit to analyze the techniques, we prepared some information for you.
In our study we roughly distinguish between the most used techniques in competitive volleyball, which are the **bow and arrow** and the **circular technique**.
Further descriptions and visual representation on the next page.

Your expertise and observations are instrumental in the success of this study, providing key insights that will enhance prevention in shoulder injuries.
Thank you for your support in analyzing the spiking techniques of your players!

TECHNIQUE DESCRIPTION

UNI GRAZ

BOW AND ARROW

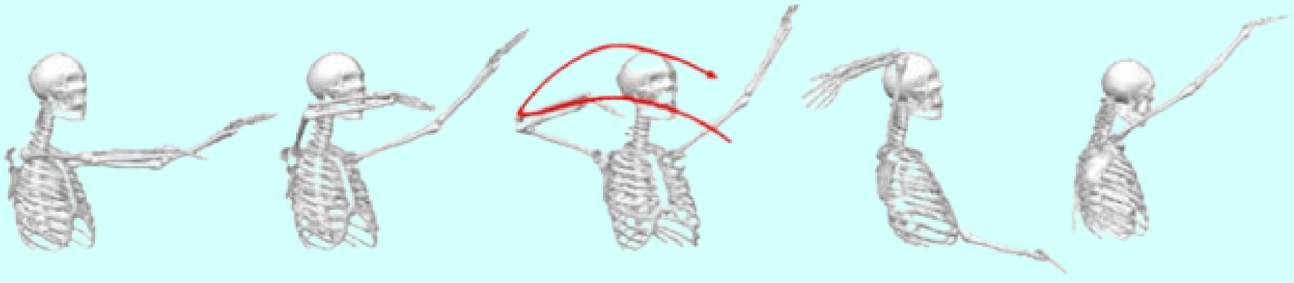

CIRCULAR

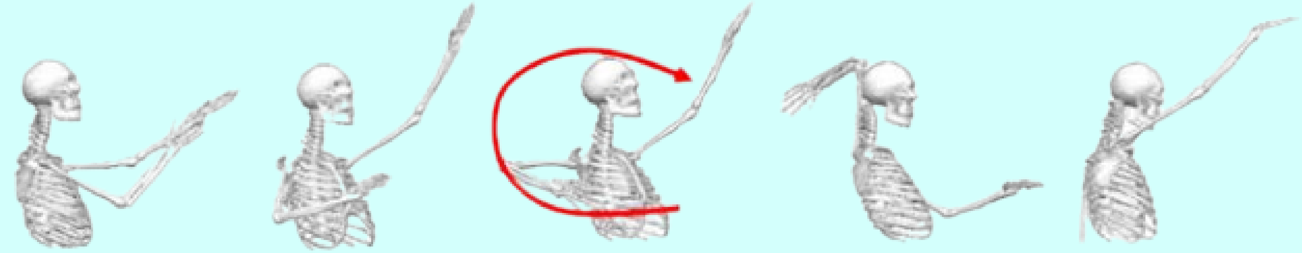

I II III IV V

BOW AND ARROW	CIRCULAR
INITIATION OF THE COCKING PHASE (I):	
Wrist above shoulder; elbow above or at the shoulder height	Wrist below or at the same height or above shoulder; elbow below or at the same height with shoulder
WIND-UP PHASE (II):	
Elbow above or at the same height with shoulder; wrist between forehead and shoulder height	Elbow and wrist at the same height or below shoulder
FINAL COCKING POSITION (III):	
Elbow above or at the same height with shoulder; wrist between forehead and shoulder height	Elbow and wrist at the same height or below shoulder
