# Supplementary material for: The association between spike technique and injuries in competitive volleyball players—a pilot study
Source: Front Sports Act Living. 2026 Feb 3;8:1737436. doi: 10.3389/fspor.2026.1737436 (PMC12909513; doi:10.3389/fspor.2026.1737436)
Supplement: Supplementary file 2 [file Datasheet2.pdf]

Supplementary Material

1 Questionnaire used in the online survey

Dear Participant,

Thank you for taking the time to complete this questionnaire. Your input is incredibly valuable to us!

This study forms an integral part of an exciting international collaboration aimed at exploring the link between spiking techniques and shoulder injuries in competitive volleyball.

Your participation is completely voluntary, and rest assured, all responses will remain anonymous and confidential. The survey is designed to be quick and easy, taking only about 10 minutes of your time.

Thank you for contributing to this research. Your insights will help shape the future of volleyball training and injury prevention.

Best regards,

Carmen Pusch, BSc University of Graz Institute of Human Movement Science, Sports and Health In cooperation with -Markus Tilp (Institue of Human Movement Science, Sport and Health, Karl Franzens University of Graz) -Karen Zentraf (Department of Movement Science and Training in Sports, Goethe University Frankfurt) -George Giatsis (Department of Physical Education and Sports Science, Aristotle University of Thessaloniki, Greece) -Alexandre Medeiros (Institute of Physical Education and Sports, Federal University of Ceará, Brasil) -Isaac Kneubuhl (TORQ Volleyball, USA)

Section A: General questions

A1. What is your nationality?

- Afghanistan ☐
- Albania ☐
- Algeria ☐
- Andorra ☐
- Angola ☐
- Antigua and Barbuda ☐
- Argentina ☐

▼

LimeSurvey

Armenia ☐

Australia ☐

Austria ☐

Azerbaijan ☐

Bahamas ☐

Bahrain ☐

Bangladesh ☐

Barbados ☐

Belarus ☐

Belgium ☐

Belize ☐

Benin ☐

Bhutan ☐

Bolivia ☐

Bosnia and Herzegovina ☐

Botswana ☐

Brazil ☐

Brunei ☐

Bulgaria ☐

Burkina Faso ☐

Burundi ☐

Cabo Verde ☐

Cambodia ☐

Cameroon ☐

Canada ☐

Central African Republic ☐

Chad ☐

Chile ☐

China ☐

LimeSurvey

- [ ] Colombia
- [ ] Comoros
- [ ] Congo (Democratic Republic)
- [ ] Congo (Republic)
- [ ] Costa Rica
- [ ] Croatia
- [ ] Cuba
- [ ] Cyprus
- [ ] Czech Republic
- [ ] Denmark
- [ ] Djibouti
- [ ] Dominica
- [ ] Dominican Republic
- [ ] Ecuador
- [ ] Egypt
- [ ] El Salvador
- [ ] Equatorial Guinea
- [ ] Eritrea
- [ ] Estonia
- [ ] Eswatini
- [ ] Ethiopia
- [ ] Fiji
- [ ] Finland
- [ ] France
- [ ] Gabon
- [ ] Gambia
- [ ] Georgia
- [ ] Germany
- [ ] Ghana

- Greece ☐
- Grenada ☐
- Guatemala ☐
- Guinea ☐
- Guinea-Bissau ☐
- Guyana ☐
- Haiti ☐
- Honduras ☐
- Hungary ☐
- Iceland ☐
- India ☐
- Indonesia ☐
- Iran ☐
- Iraq ☐
- Ireland ☐
- Israel ☐
- Italy ☐
- Jamaica ☐
- Japan ☐
- Jordan ☐
- Kazakhstan ☐
- Kenya ☐
- Kiribati ☐
- Kuwait ☐
- Kyrgyzstan ☐
- Laos ☐
- Latvia ☐
- Lebanon ☐
- Lesotho ☐

LimeSurvey

- Liberia ☐
- Libya ☐
- Liechtenstein ☐
- Lithuania ☐
- Luxembourg ☐
- Madagascar ☐
- Malawi ☐
- Malaysia ☐
- Maldives ☐
- Mali ☐
- Malta ☐
- Marshall Islands ☐
- Mauritania ☐
- Mauritius ☐
- Mexico ☐
- Micronesia ☐
- Moldova ☐
- Monaco ☐
- Mongolia ☐
- Montenegro ☐
- Morocco ☐
- Mozambique ☐
- Myanmar ☐
- Namibia ☐
- Nauru ☐
- Nepal ☐
- Netherlands ☐
- New Zealand ☐
- Nicaragua ☐

LimeSurvey

Niger ☐

Nigeria ☐

North Korea ☐

North Macedonia ☐

Norway ☐

Oman ☐

Pakistan ☐

Palau ☐

Panama ☐

Papua New Guinea ☐

Paraguay ☐

Peru ☐

Philippines ☐

Poland ☐

Portugal ☐

Qatar ☐

Romania ☐

Russia ☐

Rwanda ☐

Saint Kitts and Nevis ☐

Saint Lucia ☐

Saint Vincent and the Grenadines ☐

Samoa ☐

San Marino ☐

Sao Tome and Principe ☐

Saudi Arabia ☐

Senegal ☐

Serbia ☐

Seychelles ☐

LimeSurvey

Sierra Leone ☐
Singapore ☐
Slovakia ☐
Slovenia ☐
Solomon Islands ☐
Somalia ☐
South Africa ☐
South Korea ☐
South Sudan ☐
Spain ☐
Sri Lanka ☐
Sudan ☐
Suriname ☐
Sweden ☐
Switzerland ☐
Syria ☐
Tajikistan ☐
Tanzania ☐
Thailand ☐
Timor-Leste ☐
Togo ☐
Tonga ☐
Trinidad and Tobago ☐
Tunisia ☐
Turkey ☐
Turkmenistan ☐
Tuvalu ☐
Uganda ☐
Ukraine ☐

United Arab Emirates ☐
United Kingdom ☐
United States ☐
Uruguay ☐
Uzbekistan ☐
Vanuatu ☐
Vatican City ☐
Venezuela ☐
Vietnam ☐
Yemen ☐
Zambia ☐
Zimbabwe ☐

A2. Where do you live?

Afghanistan ☐
Albania ☐
Algeria ☐
Andorra ☐
Angola ☐
Antigua and Barbuda ☐
Argentina ☐
Armenia ☐
Australia ☐
Austria ☐
Azerbaijan ☐
Bahamas ☐
Bahrain ☐
Bangladesh ☐
Barbados ☐
Belarus ☐
Belgium ☐

LimeSurvey

Belize
Benin
Bhutan
Bolivia
Bosnia and Herzegovina
Botswana
Brazil
Brunei
Bulgaria
Burkina Faso
Burundi
Cabo Verde
Cambodia
Cameroon
Canada
Central African Republic
Chad
Chile
China
Colombia
Comoros
Congo (Democratic Republic)
Congo (Republic)
Costa Rica
Croatia
Cuba
Cyprus
Czech Republic
Denmark

LimeSurvey

Djibouti ☐
Dominica ☐
Dominican Republic ☐
Ecuador ☐
Egypt ☐
El Salvador ☐
Equatorial Guinea ☐
Eritrea ☐
Estonia ☐
Eswatini ☐
Ethiopia ☐
Fiji ☐
Finland ☐
France ☐
Gabon ☐
Gambia ☐
Georgia ☐
Germany ☐
Ghana ☐
Greece ☐
Grenada ☐
Guatemala ☐
Guinea ☐
Guinea-Bissau ☐
Guyana ☐
Haiti ☐
Honduras ☐
Hungary ☐
Iceland ☐

LimeSurvey

- India ☐
- Indonesia ☐
- Iran ☐
- Iraq ☐
- Ireland ☐
- Israel ☐
- Italy ☐
- Jamaica ☐
- Japan ☐
- Jordan ☐
- Kazakhstan ☐
- Kenya ☐
- Kiribati ☐
- Kuwait ☐
- Kyrgyzstan ☐
- Laos ☐
- Latvia ☐
- Lebanon ☐
- Lesotho ☐
- Liberia ☐
- Libya ☐
- Liechtenstein ☐
- Lithuania ☐
- Luxembourg ☐
- Madagascar ☐
- Malawi ☐
- Malaysia ☐
- Maldives ☐
- Mali ☐

LimeSurvey

Malta ☐
Marshall Islands ☐
Mauritania ☐
Mauritius ☐
Mexico ☐
Micronesia ☐
Moldova ☐
Monaco ☐
Mongolia ☐
Montenegro ☐
Morocco ☐
Mozambique ☐
Myanmar ☐
Namibia ☐
Nauru ☐
Nepal ☐
Netherlands ☐
New Zealand ☐
Nicaragua ☐
Niger ☐
Nigeria ☐
North Korea ☐
North Macedonia ☐
Norway ☐
Oman ☐
Pakistan ☐
Palau ☐
Panama ☐
Papua New Guinea ☐

LimeSurvey

- Paraguay ☐
- Peru ☐
- Philippines ☐
- Poland ☐
- Portugal ☐
- Qatar ☐
- Romania ☐
- Russia ☐
- Rwanda ☐
- Saint Kitts and Nevis ☐
- Saint Lucia ☐
- Saint Vincent and the Grenadines ☐
- Samoa ☐
- San Marino ☐
- Sao Tome and Principe ☐
- Saudi Arabia ☐
- Senegal ☐
- Serbia ☐
- Seychelles ☐
- Sierra Leone ☐
- Singapore ☐
- Slovakia ☐
- Slovenia ☐
- Solomon Islands ☐
- Somalia ☐
- South Africa ☐
- South Korea ☐
- South Sudan ☐
- Spain ☐

LimeSurvey

- Sri Lanka ☐
- Sudan ☐
- Suriname ☐
- Sweden ☐
- Switzerland ☐
- Syria ☐
- Tajikistan ☐
- Tanzania ☐
- Thailand ☐
- Timor-Leste ☐
- Togo ☐
- Tonga ☐
- Trinidad and Tobago ☐
- Tunisia ☐
- Turkey ☐
- Turkmenistan ☐
- Tuvalu ☐
- Uganda ☐
- Ukraine ☐
- United Arab Emirates ☐
- United Kingdom ☐
- United States ☐
- Uruguay ☐
- Uzbekistan ☐
- Vanuatu ☐
- Vatican City ☐
- Venezuela ☐
- Vietnam ☐
- Yemen ☐

LimeSurvey

Zambia ☐

Zimbabwe ☐

A3. **What is your gender?**

FEMALE ☐

MALE ☐

OTHER ☐

A4. **What is your date of birth?**

A5. **What is your weight?**

A6. **Which units do you use for weight?**

kg ☐

lbs ☐

A7. **What is your height?**

A8. **Which units do you use for height?**

cm ☐

feet & inch (e.g. 5'3") ☐

A9. **How long do you already play volleyball?**

A10.

Do you know the different techniques of the spike as shown in the picture?

Picture 1 shows the circular technique.

Picture 2 shows the bow and arrow (low) technique.

Yes ☐

No ☐

LimeSurvey

Section B: Volleyball-specific-questions

B1. **Please ask your coach to answer this question.**

Which technique do you use?

- Picture 1: Circular technique ☐
- Picture 2: Bow and arrow (low) technique ☐
- None of these ☐

B2. **Did you change your spiking technique due to an injury?**

- YES ☐
- NO ☐

B3. **In which volleyball league do you play?**

- national/regional ☐
- international ☐
- Other ☐

Other

LimeSurvey

B4. What position do you play?

If you changed your position please mention that in the comment-box.

- setter ☐
- libero ☐
- middle blocker ☐
- outside hitter/swing hitter ☐
- opposite hitter ☐

Section C: Shoulder Injuries

Injury means it stopped you from training/playing Volleyball.

C1. Did/do you have any injurys concerning your shoulder?

- YES ☐
- NO ☐

Section D: Shoulder Injury 1

D1. Type of shoulder injury:

If your injury is not listed, please use the comment box.

- Impingement ☐
- Labral Tears (SLAP-lesion) ☐
- Biceps Tendonitis ☐
- Shoulder Instability ☐
- Rotator Cuff Injury ☐
- other (->comment box) ☐

D2. **Date of shoulder injury:**

D3. **Was surgery needed?**

Yes ☐
No ☐

D4. **Duration of training downtime (in weeks)**

D5. **Duration of competition downtime (in weeks)**

D6. **Do you want to add another shoulder injury?**

YES ☐
NO ☐

Section E: Shoulder Injury 2

E1. **Type of shoulder injury:**

If your injury is not listed, please use the comment box.

Impingement ☐
Labral Tears (SLAP-lesion) ☐
Biceps Tendonitis ☐
Shoulder Instability ☐
Rotator Cuff Injury ☐
other (->comment box) ☐

E2. **Date of shoulder injury:**

E3. **Was surgery needed?**

Yes ☐
No ☐

LimeSurvey

E4. **Duration of training downtime (in weeks)**

E5. **Duration of competition downtime (in weeks)**

E6. **Do you want to add another shoulder injury?**

YES ☐

NO ☐

Section F: Shoulder Injury 3

F1. **Type of shoulder injury:**

If your injury is not listed, please use the comment box.

Impingement ☐

Labral Tears (SLAP-lesion) ☐

Biceps Tendonitis ☐

Shoulder Instability ☐

Rotator Cuff Injury ☐

other (->comment box) ☐

F2. **Date of shoulder injury:**

F3. **Was surgery needed?**

Yes ☐

No ☐

F4. **Duration of training downtime (in weeks)**

F5. **Duration of competition downtime (in weeks)**

LimeSurvey

F6. **Do you want to add another shoulder injury?**

YES ☐

NO ☐

Section G: Shoulder Injury 4

G1. **Type of shoulder injury:**

If your injury is not listed, please use the comment box.

Impingement ☐

Labral Tears (SLAP-lesion) ☐

Biceps Tendonitis ☐

Shoulder Instability ☐

Rotator Cuff Injury ☐

other (->comment box) ☐

G2. **Date of shoulder injury:**

G3. **Was surgery needed?**

Yes ☐

No ☐

G4. **Duration of training downtime (in weeks)**

G5. **Duration of competition downtime (in weeks)**

Section H: Shoulder-related symptoms

Symptoms means anything (e.g. pain), that affected you training/playing Volleyball.

H1. **Did/do you have any symptoms/problems concerning your shoulder?**

YES ☐

NO ☐

LimeSurvey

Section I: Shoulder-related symptom 1

I1. Type of symptom:

If your symptom is not listed, please use the comment box.

- Pain ☐
- Clicking ☐
- Stiffness ☐
- Swelling ☐
- Loss of strength ☐
- other (-> comment box) ☐

I2. Since when do you have the symptom?

I3. How does this symptom affect your performance in volleyball?

I4. Do you want to add another symptom?

- YES ☐
- NO ☐

LimeSurvey

Section J: Shoulder-related symptom 2

J1. **Type of symptom:**

If your symptom is not listed, please use the comment box.

- Pain ☐
- Clicking ☐
- Stiffness ☐
- Swelling ☐
- Loss of strength ☐
- other (-> comment box) ☐

J2. **Since when do you have the symptom?**

J3. **How does this symptom affect your performance in volleyball?**

J4. **Do you want to add another symptom?**

- YES ☐
- NO ☐

LimeSurvey

Section K: Shoulder-related symptom 3

K1. Type of symptom:

If your symptom is not listed, please use the comment box.

- Pain ☐
- Clicking ☐
- Stiffness ☐
- Swelling ☐
- Loss of strength ☐
- other (-> comment box) ☐

K2. Since when do you have the symptom?

K3. How does this symptom affect your performance in volleyball?

Section L: Prevention

L1. Do you follow a specific training routine for your shoulders?

- YES ☐
- NO ☐

L2. Do you take pain killers to tolerate training?

- YES ☐
- NO ☐

LimeSurvey

L3. Do you use kinesiotape?

YES ☐

NO ☐

Dear Participant,

thank you for completing this questionnaire. For questions or other inquiries, please contact me.

Best regards,

Carmen Pusch, BSc carmen.pusch@edu.uni-graz.at University of Graz Institute of Human Movement Science, Sports and Health In cooperation with: Markus Tilp (Institue of Human Movement Science, Sport and Health, Karl Franzens University of Graz) Karen Zentraf (Department of Movement Science and Training in Sports, Goethe University Frankfurt) George Giatsis (Department of Physical Education and Sports Science, Aristotle University of Thessaloniki, Greece) Alexandre Medeiros (Institute of Physical Education and Sports, Federal University of Ceará, Brasil) Isaac Kneubuhl (TORQ Volleyball, USA)
